# The Role of Left Ventricular Ejection Fraction and Left Ventricular Outflow Tract Velocity-Time Integral in Assessing Cardiovascular Impairment in Septic Shock

**DOI:** 10.3390/jpm12111786

**Published:** 2022-10-29

**Authors:** Konstantinos Spathoulas, Vasiliki Tsolaki, George E. Zakynthinos, Dimitrios Karelas, Demosthenes Makris, Epaminondas Zakynthinos, John Papanikolaou

**Affiliations:** 1 Department of Cardiology, General Hospital of Trikala, 42100 Trikala, Greece; 2 Department of Critical Care, School of Medicine, University of Thessaly, University Hospital of Larissa, 41110 Thessaly, Greece

**Keywords:** left ventricular ejection fraction (LVEF), velocity-time integral (VTI), cardiac output, septic shock, septic cardiomyopathy, vasoplegia

## Abstract

Background: the role of echocardiography in septic shock remains controversial, since depressed cardiac afterload may overestimate left ventricular (LV) systolic performance and mask septic cardiomyopathy (SC). We hypothesized that afterload-adjusted LV ejection fraction (LVEF) and LV outflow tract velocity-time integral (VTI) values for given systemic vascular resistances (SVR) could provide novel insights into recognizing and stratifying the severity of SC. Methods: in this observational, monocentric study, we prospectively included 14 mechanically-ventilated patients under septic-shock who all had a Pulse index Continuous Cardiac Output (PiCCO) system in place for hemodynamic monitoring. Echocardiographic and PiCCO longitudinal examinations (71 measurements overall) were performed simultaneously at the onset of septic shock and every 12 h for 60 h overall. Results: VTI-derived stroke volume (SV) and cardiac output (CO) were significantly correlated with PiCCO measurements (r ≥ 0.993, both *p* < 0.001). LVEF and VTI showed linear and exponential inverse correlation to SVR (R2 = 0.183 vs. 0.507 and *p* < 0.001 vs. *p* < 0.001, respectively). The equations LVEF = 86.168 − 0.011 × SVR and VTI = 41.23 × e^(−0.0005×SVR)^ were found to provide “predicted” values for given SVR. Measured to predicted LVEF ratios (for given SVR), the afterload-adjusted LVEF defined the severity of SC (mild ≥ 90%, 80% ≤ moderate < 90% and severe < 80%). Mild SC demonstrated normal/supra-normal LVEF, normal VTI and SVR. Moderate SC showed lower LVEF and SVR, yet increased LV end-diastolic volume (LVEDV), VTI, SV and CO compared with mild SC (all *p* < 0.05). Severe SC was distinguished from moderate SC by markedly reduced LVEF, LVEDV, VTI, SV, CO and significantly increased SVR (all *p* < 0.05). LVEF and VTI decreased over time in mild SC, LVEF decreased in moderate SC, and LVEF and VTI increased over time in severe SC (*p* ≤ 0.038). LVEF and VTI demonstrated significant performance in identifying severe SC [cut-off < 61.5%, area under the curve (AUC) = 1 ± 0.0, sensitivity/specificity = 100/100, *p* < 0.001 vs. cut-off < 17.9 cm, AUC = 0.882 ± 0.042, sensitivity/specificity = 80/77, *p* < 0.001, respectively]. VTI but not LVEF demonstrated significant diagnostic performance in identifying both SVR < 800 dynes·s·cm^−5^ and SVR > 1500 dynes·s·cm^−5^ (cut-off > 24.46 cm, AUC = 0.889 ± 0.049, sensitivity/specificity = 75/100, *p* < 0.001; cut-off < 16.8, AUC = 0.0.857 ± 0.082, sensitivity/specificity = 83/86, *p* = 0.002, respectively).Conclusions: our study suggests that ICU bedside echocardiographic assessment of LVEF, VTI and their adjusted to corresponding SVR values provides valuable insights for the comprehension of SC phenotypes, underlying vasoplegia and cardiac output fluctuations in septic shock.

## 1. Introduction

Bedside echocardiography has become the standard of care in assessing critically ill patients with hemodynamic compromise [1,2]. It represents a versatile and reliable hemodynamic bedside imaging tool in assessing stroke volume (SV) and cardiac output (CO), valvular pressure gradients, dynamic LV outflow tract (LVOT) gradient, LV filling pressures, hypovolemia and right heart pressures [3]. In this respect, echocardiography can provide valuable information regarding the underlying etiology of shock. Consequently, it is a precious means not only in reaching a diagnosis but also in guiding critical therapeutic decisions (i.e., fluid challenges, inotropic support, pericardiocentesis, thrombolysis, percutaneous coronary intervention). However, the diagnostic performance of echocardiography in the setting of septic shock still remains controversial.

In septic shock states, endotoxemia and over-production of inflammatory mediators not only may cause vasoplegia and prolonged vascular failure, but also myocardial dysfunction also known as septic cardiomyopathy [4,5,6]. Vasoplegia may result in critically reduced systemic vascular resistances (SVR) and decreased cardiac afterload masking depressed intrinsic myocardial contractility. Even a severely diseased heart may be capable of pumping a seemingly normal SV and CO, as so in septic shock normal CO values cannot rule out LV dysfunction.

Two of the most commonly used echocardiographic parameters in the assessment of LV systolic function include LV ejection fraction (LVEF) and LVOT velocity-time integral (VTI). Both indices have been extensively validated in diseased hearts [7,8], while LVOT VTI is an established marker of successful volume responsiveness after fluid loading or passive leg raising in hemodynamically compromised patients [9,10,11]. In addition, both LVEF and LVOT VTI have been used for prognostic reasons in sepsis with studies reporting that lower LVEFs [12] but higher VTIs [13] are associated with increased survival. Nevertheless, reduced cardiac afterload in septic shock may result in pseudo-normal LVEF and VTI values [14,15]. Under such circumstances, the latter reflect decreased arterial tone rather than intrinsic LV contractility [16]. Accordingly, echocardiography is likely to underestimate cardiac dysfunction in septic shock [14,17]. Given that reference values for LVEF and VTI are standardized for normal afterload [14], the role of these indices in septic shock still remains challenging.

This prospective study aimed to evaluate the correlation between LVEF and LVOT VTI to SVR values as assessed by Pulse index Continuous Cardiac Output (PiCCO) thermodilution method, seeking for “reference” or “predicted” echocardiographic values for given SVR in septic shock. We hypothesized that afterload-adjusted echocardiographic indices (measured to predicted LVEF and VTI ratios) could provide valuable insights into recognizing and classifying the severity of SC. Secondarily, we evaluated the diagnostic performance of echocardiography in predicting PiCCO-derived SV and CO in several stages and loading conditions. We finally examined the diagnostic performance of conventional echocardiographic indices (principally LVEF and VTI measurements) in identifying noninvasively the severity of cardiovascular impairment in septic shock.

## 2. Methods

### 2.1. Patient Population

All consecutive adult patients admitted in the twelve-bed medical-surgical Intensive Care Unit (ICU) of a Greek university hospital (University Hospital of Larisa, Thessaly, Greece) between 1 June 2018 and 31 May 2019, were prospectively screened for eligibility. Inclusion criteria included: (1) age ≥ 18 years old, (2) need for mechanical ventilation, (3) diagnosis of septic shock according to the 2012 Surviving Sepsis Campaign criteria [18] and (4) PiCCO system in place for monitoring purposes. Exclusion criteria were: (1) pregnancy, (2) heart rhythm other than sinus rhythm at admission, (3) known history of coronary artery disease or heart failure with reduced LVEF, (4) aortic stenosis and (5) moderate-to-severe aortic valve regurgitation or subaortic obstruction (fixed or dynamic), both of which produce high LVOT velocities and overestimate VTI.

Patients were clinically assessed and managed according to the standard protocols of care [18,19,20] (see also Supplemental file S1, Clinical assessment). Clinical management decisions were made by the attending physicians, including the intention to place a PiCCO system for diagnosis or monitoring as part of standard care. All cases were discussed daily in a multidisciplinary meeting.

The sample size of our study was selected on the basis of previously reported data which suggested a marked heterogeneity in the prevalence of SC regarding the timing of echocardiography, ranging from only 18% in patients assessed early after the onset of septic shock to 70% after serial echocardiographic measurements within a 3-day period [21,22]. Thus, prospective power calculation was performed aiming to depict a similar increase (388%) in the prevalence of SC over time, with a probability of a type-I error of 5% and a power of 80%, and revealed that at least 14 patients would be required for our longitudinal analysis.

The study protocol was approved by the Internal Review Board and Ethics Committee of the University of Thessaly (ID: 23753; 19 April 2018) and conformed to the ethical guidelines of the 1975 Declaration of Helsinki. Patients’ next of kin provided informed consent for all participants.

### 2.2. Study Endpoints

The main purpose of the study was to investigate for “reference” or “predicted” echocardiographic values for given SVR in septic shock. We hypothesized that afterload-adjusted LVEF and VTI values (measured to predicted ratios) could provide additional information in the diagnosis of SC and stratification of its severity. Secondarily, we evaluated the diagnostic performance of conventional echocardiography in assessing noninvasively (a) hemodynamics in several stages and loading conditions, and (b) the severity of cardiovascular impairment in septic shock.

### 2.3. Transthoracic Echocardiography (TTE) Examination and PiCCO Measurements

TTE and PiCCO examinations were performed every 12 h for 60 h or until death or discharge from the ICU (a maximum of six studies for each patient enrolled). Echocardiographic and PiCCO measurements were carried out sequentially. Fluid, inotrope and vasopressor infusion rate was exactly the same for each set of hemodynamic and echocardiographic measurements.

i. TTE measurements

Echocardiography (Phillips iE33, Andover, MA, USA) was performed by two experienced echocardiographers (J.P., E.Z.). LVEF was measured by the two-dimensional Simpson’s biplane method of disks, LVOT VTI from the apical five chamber view, and LVOT diameter for VTI-derived SV and CO was measured from the parasternal long axis view, as recommended [23,24,25,26,27] (see also Supplemental file S1, TTE measurements).

ii. PiCCO measurements

The PiCCO system (Pulsiocath 5F, 20 cm, PV2015L20; Pulsion Medical Systems AG, Munich, Germany) was used for hemodynamic measurements [including CO, SVR, extravascular lung water (EVLW) and central venous pressure (CVP)] [28,29] (see also Supplemental file S1, PiCCO measurements). Physicians who performed the echocardiographic examination were unaware of the results of the thermodilution study.

### 2.4. Mathematical Calculation Models for Afterload-Adjusted LVEF and LVOT VTI Values

i. LVEF plotted against SVR; introduction of the novel index afterload-adjusted LVEF (aLVEF).

**Figure 1:**
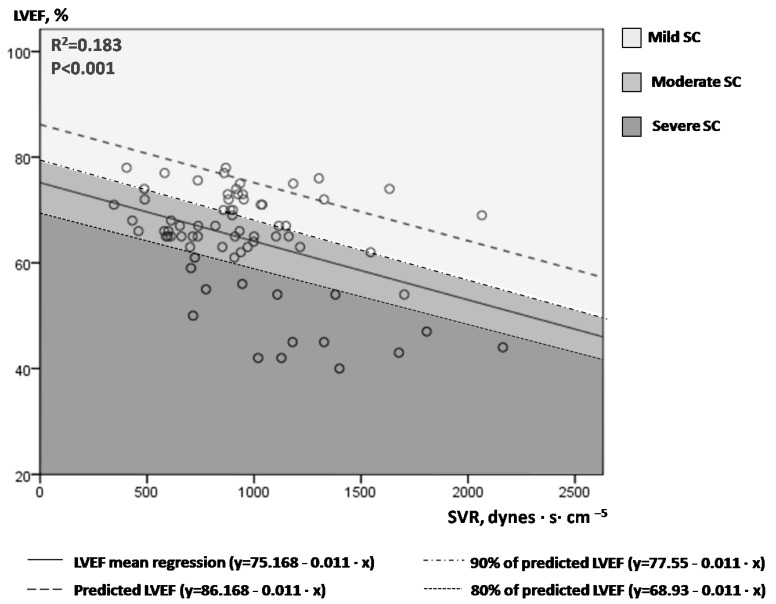
Correlation of serial LVEF and SVR measurements in our 14 patients with septic shock.

Figure 1 illustrates LVEF values plotted against the corresponding SVR values in the 71 measurements performed overall in our study population (14 patients). A linear mean regression line (continuous line, Figure 1) fitted best to predict mean LVEF by SVR values [line equation: LVEF = 75.168 − 0.011 × SVR, R^2^ = 0.183, *p* < 0.001)]. For each SVR, we hypothesized that the highest LVEF values may suggest normal/near normal cardiac function, while the lowest LVEF values may indicate depressed LV contractile function. The upper limit of the 80% prediction interval of the regression line (dotted line, Figure 1) was used to define the “normal” predicted LVEF for given SVR values [line equation: LVEF = 86.168 − 0.011 × SVR)]. For example, for SVR values of 500, 1000 and 1500 dynes·s·cm^−5^, the “predicted” LVEF values are 80.67%, 75.17% and 69.67%, respectively. Next, we introduced the index afterload-adjusted LVEF (aLVEF) in each specific patient as a marker of septic cardiomyopathy, which was calculated as follows:

aLVEF (%) = LVEF_measured_/LVEF_predicted_ × 100%

ii. VTI plotted against SVR; introduction of the novel index afterload-adjusted VTI (aVTI).

**Figure 2:**
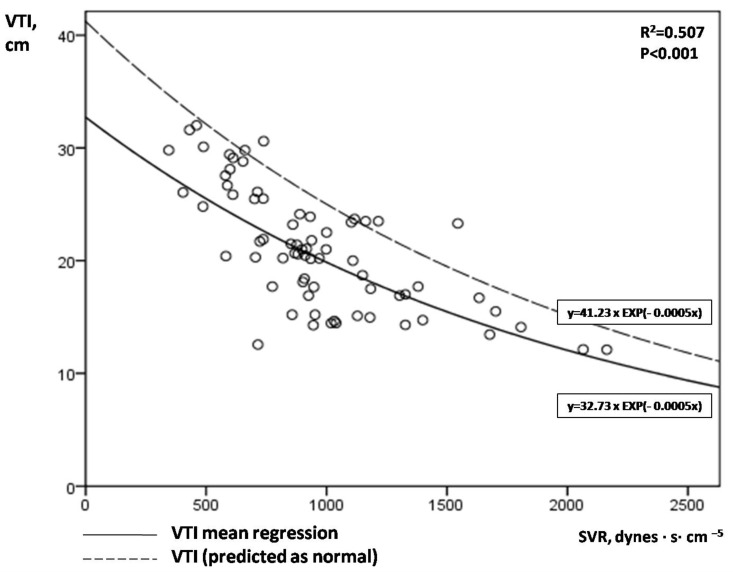
Correlation of serial VTI and SVR measurements in our 14 patients with septic shock.

Figure 2 illustrates the correlation between aortic VTI and SVR. An exponential mean regression model [continuous line: VTI = 32.73 × EXP(−0.0005 × SVR) or VTI = 32.73 × e^(−0.0005×SVR)^, R^2^ = 0.507, *p* < 0.001] fitted best to predict VTI by SVR values. The upper limit of the 80% prediction interval of this exponential mean regression line [dotted line: VTI = 41.23 × EXP(−0.0005 × SVR)] defined the “normal” or “predicted” VTI for each SVR value. We hypothesized that low VTI compared to the predicted one for a given SVR may possibly indicate cardiac inability to keep up with peripheral demands in shock. Similar to aLVEF, we introduced afterload-adjusted VTI (aVTI) as a marker of cardiac pumping ability to meet peripheral demands in shock, which was calculated as follows:

aVTI (%) = VTI_measured_/VTI_predicted_ × 100%

### 2.5. Statistical Analysis

The results are expressed as means ± standard error (SE), unless otherwise stated. Kolmogorov-Smirnov test was used for normality assessment. Chi-square or Fisher’s exact test were used to compare categorical variables and *t*-test or Man-Whitney *U* test to compare continuous variables as appropriate. Regression analysis was used to determine associations among continuous variables, to estimate the mean regression lines (linear or exponential) fitted best each time, to define the 80% upper limit of the prediction intervals of the regression lines and to produce the equation of each line. One-way analysis of variance was used for multiple comparisons. To assess differences in serial echocardiographic measurements among subgroups, mean regression lines were created and compared by using linear mixed model analysis. Receiver operating characteristic (ROC) curve analysis was performed to evaluate the diagnostic performance of echocardiography in predicting severe septic cardiomyopathy, normal afterload or vasoplegia in septic shock. The Youden’s J statistic was used for selecting the optimum cutoff point in each ROC analysis. As a basis for test decisions, a significance level of 0.05 (*p* value) was chosen. The statistical package SPSS 17.0 (SPSS Inc., Chicago, IL, USA) was used.

## 3. Results

LVEF, LVOT VTI and PiCCO-derived hemodynamics were studied in 14 consecutive patients with septic shock (71 measurement sets in total). Table 1 summarizes the clinical characteristics of patients included.

Ten patients survived the shock and each of them completed the six predetermined ultrasound/PiCCO measurements over a time period of 60 h. Four patients died due to septic shock (one on day-1, two on day-2, and one on day-3), so only one to five sets of echocardiographic and hemodynamic measurements were obtained. Table 2 illustrates the echocardiographic and hemodynamic data in each of our 14 patients examined. The four non-survivors in our cohort were characterized by significantly increased ratio of mitral inflow pulsed-wave Doppler E-wave velocity to tissue Doppler imaging Em velocity at the lateral mitral annulus (E/Em) and CVP values, and markedly decreased central venous oxygen saturation (ScvO_2_) values on presentation (8.9 ± 1 vs. 7.3 ± 0.2, *p* = 0.041; 10 ± 1.7 vs. 6.3 ± 0.8, *p* = 0.045; and 64.1 ± 6.4 vs. 76.6 ± 1.6, *p* = 0.019, respectively). Instead, initial LVEF, VTI, minute distance, SVR, noradrenaline dose and EVLW measurements showed no deference between survivors and non-survivors (all *p* ≥ 0.217).

Significant correlations between the cardiac and hemodynamic variables examined are presented in Supplemental Table S1. Interestingly, VTI correlated better than LVEF with SV (*r* = 0.910, *p* < 0.001 vs. *r* = 0.391, *p* = 0.001), CO (*r* = 0.743, *p* < 0.001 vs. *r* = 0.514, *p* < 0.001) and SVR (*r* = −0.702, *p* < 0.001 vs. *r* = −0.428, *p* < 0.001). In addition, ScvO_2_ correlated positively with VTI, SV and CO (*r* ≥ 0.391, *p* ≤ 0.015) but negatively with SVR (*r* = −0.486, *p* = 0.002). EVLW, an index of pulmonary edema in sepsis, showed no correlation with any parameter examined.

Decreased aVTI was significantly correlated with increased E/Em and depressed CVP values (*r* = −0.318, *p* < 0.001 and *r* = 0.251, *p* = 0.035, respectively).

Figure 1. One to six times at each patient, LVEF and SVR measurements were performed simultaneously during the course of every septic shock episode (71 sets of measurements overall). An inverse correlation of LVEF and SVR was observed, with a large variation of LVEF values for a given SVR. The continuous line represents the mean regression line fitted better in our model (LVEF = 75.168 − 0.011 × SVR), while the dashed line the upper 80% of the prediction interval of the mean regression line (LVEF = 86.168−0.011×SVR). Different colored-fields discriminate LVEFs within the range of mild, moderate or severe SC. LVEF = left ventricular ejection fraction; SVR = systemic vascular resistance; SC = septic cardiomyopathy.

Figure 2. A clear exponential correlation of VTI and SVR can be observed, with a large variation of VTI values for a given SVR. The continuous line represents the mean regression line fitted better in our model [line: VTI = 32.73 × EXP(−0.0005 × SVR)], while the dotted line the upper limit of the 80% prediction interval of the mean regression line [dotted line: VTI = 41.23 × EXP(−0.0005 × SVR)]. VTI = velocity time integral; SVR = systemic vascular resistance.

i. The role of afterload-adjusted LVEF and VTI indices in SC severity stratification.

Given that SC is present in the whole spectrum of septic shock [6], we classified SC as mild (aLVEF ≥ 90% or measured LVEF ≥ 90% of predicted LVEF), moderate (80% ≤ aLVEF < 90%, or 80% of predicted LVEF ≤ measured LVEF < 90% of predicted LVEF) and severe (aLVEF < 80% or measured LVEF < 80% of predicted LVEF). Please notice the different colored fields in Figure 1.

Four patients (28.6%) demonstrated severe SC (aLVEF < 80%), five (35.7%) moderate SC (80% ≤ aLVEF < 90%) and five (35.7%) mild SC (aLVEF ≥ 90%) upon presentation. Nine patients remained in a stable cardiac state throughout the examination period, while five altered their SC status. Among the latter, three patients temporarily changed, and two (number#6 and number#10) constantly improved their SC class. One patient with severe SC (1/4), none of the patient with moderate SC (0/5), and three patients with mild SC (3/5) on presentation died due to septic shock (mortality of 25%, 0% and 60%, respectively; *p* = 0.108). Initial aVTI (but not aLVEF) measurements were found significantly decreased in non-survivors compared to survivors (67.1 ± 8.7% vs. 82.7 ± 2.3%; *p* = 0.031, respectively).

Table 3 illustrates the clinical, hemodynamic and echocardiographic characteristics in each group of SC. LVEF is clearly de-escalated as the degree of inflammatory myocardial depression increased. Instead, LVOT VTI and CO values (as well as minute distance, SV, LVEDV and aVTI) demonstrate a “parabolic” or “reverse U” correlation in relation to the degree of SC severity, manifesting greater values in the distribution of moderate rather than severe or mild SC (Table 3).

ii. LVOT VTI in assessing cardiac output in septic shock.

**Figure 3:**
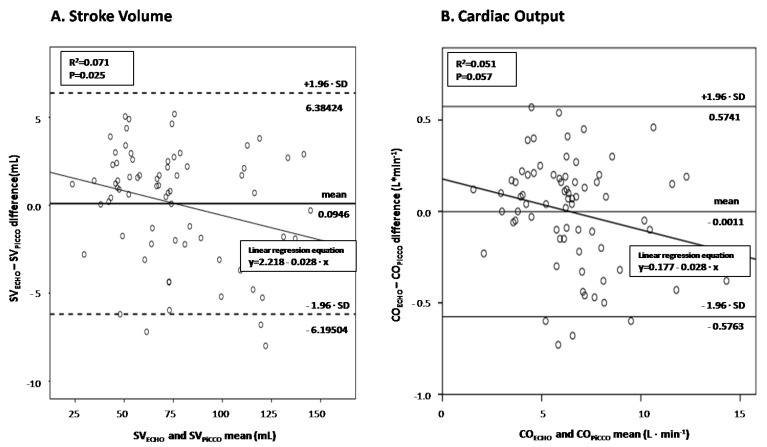
Bland and Altman plot between echocardiography-derived and PiCCO-derived SV (panel (**A**))/CO (panel (**B**)) in septic shock.

There was a significant correlation between SV measured by echocardiography and PiCCO *(r* = 0.995, *p* < 0.001), as well as between ultrasound-derived and PiCCO-derived CO (*r* = 0.993, *p* < 0.001). The diagnostic performance of LVOT VTI in predicting PiCCO-derived SV and CO was assessed by performing Bland-Altman plots (Figure 3A,B, respectively). Interestingly, echocardiographic SV significantly overestimated the corresponding hemodynamic measurements at low cardiac output states and underestimated them at high-output septic shock states (*p* = 0.025), while echocardiographic CO depicted borderline significant value (*p* = 0.057).

Figure 3. Visual inspection suggests that the echocardiographic measurements are increasingly underestimating the PiCCO-derived ones with increasing magnitude of SV (panel A) and CO (panel B) Linear regression analyses quantifying the decrease of echocardiographic minus hemodynamic differences as cardiac output increases showed significant and borderline significant proportional bias for SV and CO measurements (slopes of −0.028 in both regression lines; *p* = 0.025 and *p* = 0.057), respectively. SV = stroke volume; CO = cardiac output.

iii. Kinetics of LVEF and VTI in SC subgroups.

**Figure 4:**
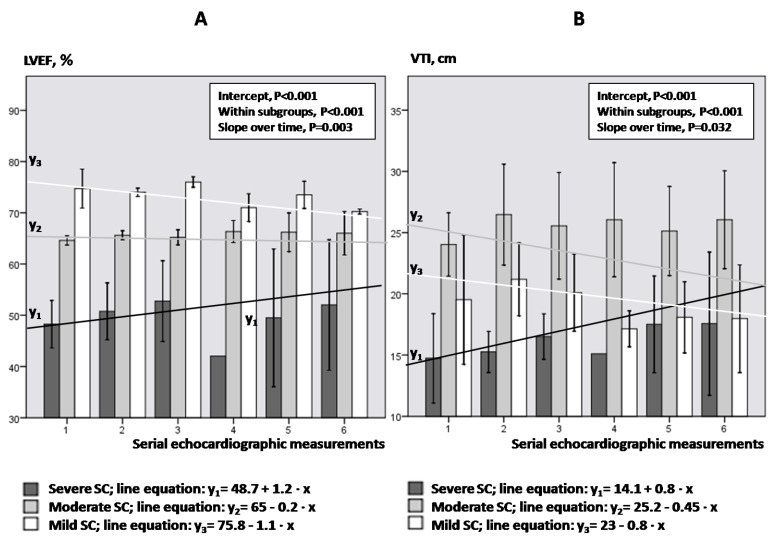
LVEF (panel (**A**)) and VTI (panel (**B**)) kinetics regarding the degree of SC.

Figure 4A,B illustrate the LVEF and VTI kinetics over time in each group of SC, respectively. LVEF decreased significantly in mild and moderate SC, while increased significantly over time in severe SC (*p* ≤ 0.011; Bonferroni post-hoc analysis). Similarly, VTI showed a significant decrease over time in mild SC and an increase over time in severe SC (*p* ≤ 0.038).

Figure 4. Bars and vertical lines indicate mean values and standard errors, respectively. Kinetics in each subgroup is indicated by the corresponding mean regression line for mild (white line), moderate (gray line) and severe SC (black line). LVEF = left ventricular ejection fraction; VTI = velocity-time integral; SE = standard error; SC = septic cardiomyopathy; Intercept of the regression line = the LVEF or VTI value where the regression line crosses the y-axis at the theoretical day = 0; Slope of the regression line = the rate at which LVEF or VTI values change between two-consequent follow-up examinations.

iv. Conventional echocardiography in assessing severe SC and abnormal SVR.

**Figure 5:**
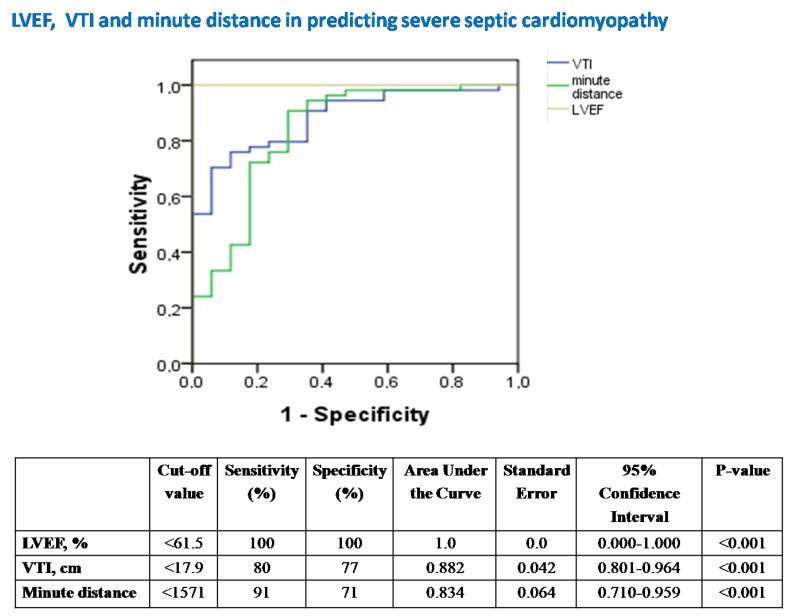
The diagnostic performance of echocardiography in predicting severe SC.

Figure 5. Receiver operator characteristic (ROC) curve analysis assessing the performance of LVEF, VTI and minute distance in predicting underlying severe SC in our 14 patients with septic shock. LVEF = left ventricular ejection fraction; VTI = velocity-time integral; SC = septic cardiomyopathy.

LVEF was the best echocardiographic marker in diagnosing severe SC (Figure 5).

**Figure 6:**
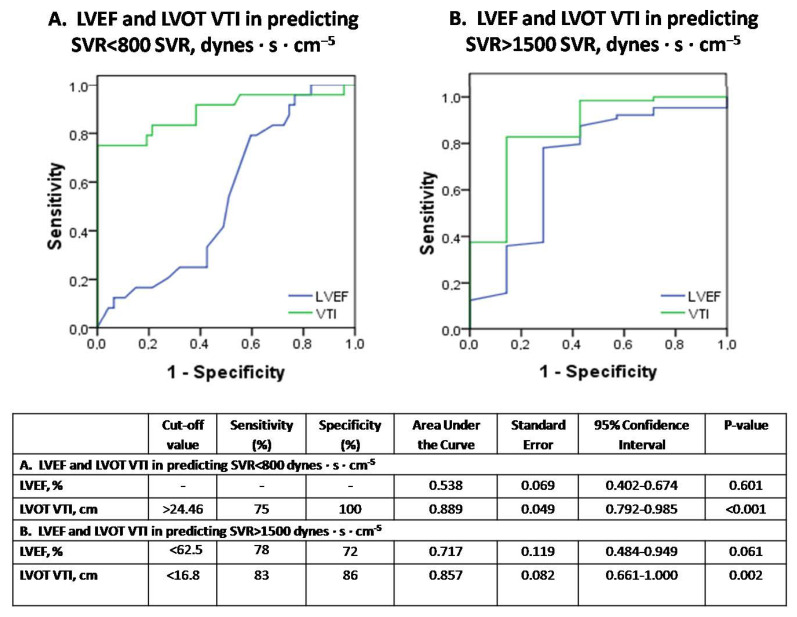
The diagnostic performance of echocardiography in predicting vasoplegia (SVR < 800 dynes·s·cm^−5^) or normal SVR (SVR > 1500 dynes·s·cm^−5^) in septic shock (subfigure panel (**A**) and (**B**), respectively).

In contrast, ROC-curves of Figure 6A,B illustrate that LVOT VTI but not LVEF demonstrates diagnostic utility in assessing both severely depressed SVRs < 800 dynes·s·cm^−5^ and increased SVRs > 1500 dynes·s·cm^−5^, respectively.

Figure 6. Receiver operator characteristic (ROC) curve analysis assessing the performance of LVEF and VTI in predicting SVR < 800 dynes·s·cm^−5^ (panel A) or SVR > 1500 dynes·s·cm^−5^ (panel B), respectively, in our 14 patients with septic shock. SVR = systemic vascular resistance; LVEF = left ventricular ejection fraction; VTI = velocity-time integral.

## 4. Discussion

In the present study, we introduced the concept of “normal” or “predicted” LVEF and LVOT VTI values for given SVR, and attempted to standardize these echocardiographic parameters of LV systolic function to the corresponding LV afterload, by adjusting the measured values each time according to the “predicted” ones for given SVR. On the basis of our analysis, we classified the severity of SC and identified the common characteristics of the mild, moderate and severe SC phenotype. However, our findings may suggest that not only afterload-adjusted, but also conventional LVEF and VTI measurements can provide valuable information about underlying SC at bedside. Furthermore, our results highlight the utilities and pitfalls of LVOT VTI in evaluating and monitoring CO fluctuations, and its possible value in diagnosing severe vasoplegia in septic shock.

The present study proposes two two-dimensional diagrams (nomograms) that allow the approximate graphical computation of “reference” or “predicted” LVEF and VTI values for given SVR values in septic shock [line equations: LVEF = 86.168 − 0.011 × SVR and VTI = 41.23 × e^(−0.0005×SVR)^, respectively] (Figure 1 and Figure 2). To our knowledge, this is unique in the literature until now. In this respect, both the severity of SC and circulatory failure can be estimated, by comparing the measured LVEF and VTI values, respectively, to the predicted ones (as the latter are assessed from our study). The conceptualization of afterload-adjusted LVEF (aLVEF) index as a surrogate of cardiac impairment, and afterload-adjusted VTI (aVTI) index as a marker of the capacity of the heart to meet peripheral demands constitute novel indices set and investigated in our study. Interestingly, depressed aVTI values were significantly correlated with LV diastolic dysfunction (increased E/Em) but not LVEF or aLVEF, possibly indicating that pump insufficiency to keep up with peripheral demands may be associated with LV diastolic but not systolic dysfunction. However, the diagnostic performance of these indices remains to be tested in larger scale studies in the future.

In the present investigation we also found that echocardiographic SV and CO measurements correlate very well with the corresponding PiCCO-derived ones. However, our echocardiographic measurements tended to overestimate the PiCCO-derived values in low cardiac output states and underestimate them in hyperdynamic (high cardiac output) states (Figure 3A,B). The clinical value of this novel information proposed herein should be also examined in the future.

Our limited data may also suggest that echocardiography can play a pivotal role towards a better comprehension of the SC phenotype (Table 3). Mild SC (defined as aLVEF ≥ 90% in our study) is characterized by increased heart rate, normal/supra-normal LVEF, normal VTI values [30] and CO, and relatively normal SVR values, possibly suggesting that the primary pathogenic mechanisms of the shock are rather non-cardiac in origin. Moderate SC (80% ≤ aLVEF < 90%) is characterized by a significant reduction in mean arterial pressure (MAP) and systemic SVRs (shock principally circulatory in origin), yet normal LVEF (although lower than that observed in mild SC). Left ventricle also dilates (increased LVEDV) [31] and concomitantly VTI, SV, minute distance and CO are also markedly increased (possibly suggesting Frank-Starling adaptations). Although our small sample size precludes safe conclusions about mortality, the favorable outcome of our moderate SC patients, may indicate a possible “protective effect” of LV dilatation, as previously underlined [12,32]. Finally, severe SC (aLVEF < 80%) is characterized not only by dramatically decreased LVEF (as expected), but also by markedly reduced LVEDV, VTI, SV, minute distance and CO compared to moderate SC. MAP is further declined, while SVR is significantly elevated compared to moderate SC, possibly indicating a type of cardiogenic rather than circulatory shock [33]. Cardiac troponin and SOFA scores were significantly increased in severe SC, indicating multi-organ dysfunction including structural myocardial injury, as previously proposed [34]. Indexed to body weight EVLW (ELWI) but not E/Em echocardiographic values were found elevated in severe SC, indicating a type of inflammatory rather than hydrostatic pulmonary edema in the “cardiogenic” subset of septic shock. Finally, B-type natriuretic peptide (BNP) values were not elevated in severe SC in our cohort, in line with our previous work suggesting that heart is not the primary source of BNP in sepsis [35].

The key-message of our report is that simple echocardiographic measurements easily assessed at bedside constitute an efficient non-invasive tool to estimate SC state and the degree of peripheral vascular collapse (Table 3). Hence, hyperdynamic LVEF but nearly normal VTI may reflect mild SC (SVRs are also approximately normal), large-sized normal left ventricles (increased LVEDV, yet normal LVEF) with increased VTI may suggest moderate SC (severe vasoplegia and depressed SVRs are likely to characterize this stage), while both depressed LVEF and VTI values should rise the suspicion of severe SC (SVRs are elevated at this stage, similarly to cardiogenic shock) and possibly positive fluid balance (as increased ELWI was found in our severe SC patients). Of course, as it was anticipated, depressed (non-afterload -adjusted) LVEFs demonstrate excellent accuracy in identifying severe SC (Figure 5). In this respect, our study provides novel insights about the role of echocardiography in the assessment of SC and septic shock.

Until now it was believed that hyperdynamic LVEF may suggest severe peripheral vasoplegia [6]. However, our results indicate that LVEF failed to detect severely depressed SVRs in septic shock. Instead, LVOT VTI demonstrated significant diagnostic performance in identifying either depressed or elevated SVR in our series. In this respect, severe vasoplegia in septic shock should be suspected by increased VTI > 24.46 cm rather than hyperdynamic LVEF values (Figure 6A,B). This may also highlight the value of VTI as a simple echocardiographic marker in early decision making (i.e., low or high VTI values may possibly indicate treatment with inotropes or vasopressors, respectively). In addition, our study provides evidence that LVEF and VTI kinetics over time provide additional information about the severity of underlying SC and the response to therapeutic interventions (Figure 4).

Our study has several limitations. First and foremost, the small sample size and the additional stratification of our patients into subgroups may have weakened the power of the study and the interpretation of the results. One could consider the study as a case series rather than an observational study. We accept this skepticism. However, our sample was selected by performing prospective power calculation on the basis of previously reported data [21,22] about the role of echocardiography timing in assessing the prevalence of SC. In addition, multiple echocardiographic examinations were performed in each patient within a 3-day period, which may have further strengthened our observations. Furthermore, the strict inclusion criteria we used led to a quite homogenous non-cardiac population, by which a smaller sample size could reach a significant level of precision [36]. Second, different isolates may have different impact on the type/severity of SC and/or vasoplegia; however, the small sample size and the multiple pathogens isolated in some patients made it difficult to draw relevant conclusions. Third, LVOT VTI is influenced by several parameters (i.e., anemia, heart rate etc.) [37] that have not been valued in the study. Whether our findings are widely applicable in septic shock is largely unknown. Nevertheless, larger-scale studies are needed to validate our results in the future.

## 5. Conclusions

In conclusion, our study highlights the importance of utilizing standardized to afterload LVEF values (corrected for given SVR) rather than simple LVEF measurements in assessing the degree of myocardial dysfunction in septic shock. Afterload-adjusted VTI values (corrected for given SVR) are also important towards a better comprehension of the detrimental pathways in sepsis. Finally, our data provide evidence that even the combination of conventional (non-afterload-standardized) LVEF and VTI measurements offer valuable insights for the specification of septic shock phenotype and the existence of underlying SC or vasoplegia, while the VTI represents a reliable marker for cardiac output monitoring.

## Figures and Tables

**Table 1:** Characteristics of each of our 14 patients included in the study.

Patient Number	Sex	Age	APACHE II	Underlying Disease	Admitting Etiology	SOFA Score	Noradren. Dose, μg/kg/min	Isolated Pathogen		Outcome of Shock Episode
								Blood Culture	Other-Site Culture	
1	M	57	18	AH	ICH	7.7 ± 0.3	0.21 ± 0.07	-	PsA (BS)	Survived
2	M	55	22	AH	ICH	10.3 ± 0.3	0.32 ± 0.05	AB	-	Survived
3 (3 meas.)	M	72	24	AH	Bladder cancer	11 ± 0	1.06 ± 0.27	CA	KP (BS)	Died (day 2)
4	M	82	19	AH, AAA	Fecal peritonitis	10.7 ± 0.3	0.09 ± 0.02	AB	AB (BS)	Survived
5	F	75	17	COPD, DM	ARDS	8 ± 0	0.55 ± 0.2	-	AB (BS)	Survived
6	M	65	15	AH	ICH	8.7 ± 0.3	0.14 ± 0.03	-	PsA (BS)	Survived
7 (5 meas.)	F	56	21	-	Meningioma post-surgery	10.3 ± 0.3	0.35 ± 0.04	PM	-	Died (day 3)
8	F	81	15	CAD, Ischemic stroke	DTAApost-surgery	8 ± 0	0.84 ± 0.12	AB	-	Survived
9	M	72	22	AH, DM	aSAH	8.3 ± 0.3	0.2 ± 0.04	AB	-	Survived
10	F	62	17	DD	Fecal peritonitis	11.3 ± 0.9	2.36 ± 0.3	Espp.	-	Survived
11	F	65	18	HL	ARDS	12 ± 0.6	1.04 ± 0.13	-	AB (BS)	Survived
12 (2 meas.)	M	82	25	AH, CAD	Pancreatitis	15	1.01	PsA	AB (PLF, PF)	Died (day 2)
13 (1 meas.)	F	78	16	Colon Cancer	Liver Abscess	14	1.76	KPC	-	Died (day 1)
14	F	67	19	Asthma	ARDS	8.7 ± 0.3	0.38 ± 0.05	-	KP	Survived

APACHE II = Acute Physiology and Chronic Health Evaluation II; SOFA = Sequential Organ Failure Assessment; AH = arterial hypertension; AAA = abdominal aortic aneurysm; COPD = chronic obstructive pulmonary disease; DM = diabetes mellitus; CAD = coronary artery disease; DD = diverticular disease; HD = Hodgkin’s lymphoma; ICH = intracerebral hemorrhage; ARDS = acute respiratory distress syndrome; DTAA = descending thoracic aortic aneurysm; aSAH = aneurysmal subarachnoid hemorrhage; PsA = pseudomonas aeruginosa; BS = bronchial secretions; AB = acinetobacter baumannii; CA = candida albicans; KP = klebsiella pneumonia; PM = proteus mirabilis; Espp. = enterobacter species; PLF = pleural fluid; PF = peritoneal fluid; KPC = klebsiella pneumoniae carbapenemase-producing.

**Table 2:** Echocardiographic and hemodynamic measurements (mean ± SE) in each of our 14 patients examined.

Patient Number	LVEF, %	VTI, cm	CO, L/min	SVR,Dynes·s·cm^−5^	EVLW, mL	ScvO_2,_ %	CVP, mmHg
1	67.7 ± 2.2	29.4 ± 1.7	10.7 ± 0.6	554 ± 79.9	742 ± 63.1	84.3 ± 1.3	8.7 ± 1.5
2	64 ± 0.6	24 ± 1.5	7.2 ± 0.1	812 ± 93.6	635 ± 82.4	70.8 ± 1	6 ± 1.1
3 (3 meas.)	53 ± 1.5	15.2 ± 1.5	4.5 ± 0.8	1064 ± 319.5	556 ± 38.4	74.9 ± 3.4	10 ± 2.5
4	73.9 ± 1.4	18.9 ± 2.2	6.4 ± 0.4	901 ± 86.7	537.3 ± 58.2	76.4 ± 1.3	5 ± 2
5	64 ± 1	23 ± 0.3	5.4 ± 0.6	1216 ± 167.1	475.3 ± 74.5	73.3 ± 1.2	9.7 ± 0.9
6	70.7 ± 4.1	21.3 ± 0.9	6.9 ± 0.4	903.7 ± 33.7	724 ± 10.1	76.6 ± 0.6	12 ± 1.1
7 (5 meas.)	73 ± 2	15.2 ± 1.6	4.1 ± 0.3	1667 ± 220.6	574.3 ± 67.4	55.6 ± 4.8	8.3 ± 2.6
8	42.3 ± 1.5	14.5 ± 0.1	3.8 ± 0.2	1248.3 ± 116.5	519.7 ± 39.9	73.8 ± 39.9	7.3 ± 0.3
9	65.3 ± 0.3	26.7 ± 0.5	7.9 ± 0.1	593 ± 9.3	520.6 ± 27.2	81.6 ± 1.3	11 ± 1
10	55 ± 6.7	17.8 ± 3.3	2.8 ± 0.7	1553 ± 314.3	990.3 ± 15.7	79.8 ± 1.7	9 ± 1.5
11	74 ± 3.5	20.4 ± 0.1	7.4 ± 0.6	756.3 ± 88.3	751.7 ± 51	81.7 ± 2.3	5 ± 1.1
12 (2 meas.)	76	25.4	13	445.5	846.5	76.7	6.5
13 (1 meas.)	73	16.9	6.9	925	462	65	7
14	58 ± 2	19.6 ± 0.6	6 ± 0.3	907.3 ± 116.6	868 ± 8.1	71.3 ± 1.3	3.7 ± 1.5

SE = standard error; LVEF left ventricular ejection fraction; VTI velocity-time integral; SVR = systemic vascular resistance; EVLW = extravascular lung water; ScvO_2_ = central venous oxygen saturation; CVP = central venous pressure.

**Table 3:** Clinical, hemodynamic and echocardiographic characteristics of each group of septic cardiomyopathy.

	Severe SC aLVEF < 80%N = 17	Moderate SC80 ≤ aLVEF < 90%N = 30	Mild SCaLVEF ≥ 90%N = 24	*p*-Value
**Clinical characteristics**				
HR, beats/minute	88. ± 3.6	78 ± 1.6	98 ± 2.9	<0.001 ^a,b,c^
MAP, mmHg	66.9 ± 3.1	78.1 ± 1.9	80.6 ± 2	<0.001 ^a,b^
SOFA score	11.2 ± 0.6	9.1 ± 0.4	9.3 ± 0.4	0.007 ^a,b^
Noradrenaline dose, μg/kg/min	0.9 ± 1.3	0.55 ± 0.13	0.57 ± 0.15	0.29
CTnI, ng/mL	1.22 ± 0.53	0.2 ± 0.07	0.41 ± 0.19	0.011 ^a,b^
BNP, pg/mL	1085 ± 284	901 ± 417	2006 ± 846	0.352
**PiCCO-derived Hemodynamics**				
Stroke volume, mL	48.3 ± 3.2	97.7 ± 5.3	65.8 ± 4.2	<0.001 ^a,c^
Cardiac Output, L/min	4.3 ± 0.4	7.5 ± 0.4	6.5 ± 0.5	<0.001 ^a,b^
SVR, dynes·s·cm^−5^	1215 ± 104	799 ± 49	990 ± 71	0.001 ^a^
EVLW, mL	718 ± 50	660 ± 29	669 ± 31	0.516
ELWI, mL/kg	10.1 ± 0.9	8 ± 0.5	8.2 ± 0.4	0.035 ^a^
ScvO2, %	76.5 ± 1.9	78.9 ± 1.4	75.4 ± 2.8	0.452
CVP, mmHg	7 ± 0.8	8.9 ± 0.5	8.2 ± 0.7	0.154
**Echocardiographic characteristics**				
VTI, cm	16 ± 0.7	25.5 ± 0.7	19 ± 0.7	<0.001 ^a,b,c^
Stroke volume, mL	47.9 ± 2.9	97 ± 5.1	67.3 ± 4	<0.001 ^a,b,c^
Cardiac Output, L/min	4.33 ± 0.3	7.5 ± 0.4	6.6 ± 0.5	<0.001 ^a,b^
Minute Distance, cm/min	1416 ± 89	1978 ± 50	1852 ± 83	<0.001 ^a,b^
aVTI, %	71.2 ± 3	92 ± 1.8	74.8 ± 2	<0.001 ^a,c^
LVEDV, mL	95.5 ± 4.8	147.2 ± 7.3	91.5 ± 5	<0.001 ^a,c^
LVEF, %	50.1 ± 1.7	65.7 ± 0.4	73.2 ± 0.6	<0.001 ^a,b,c^
E/Em	7.7 ± 0.3	7.1 ± 0.1	7.2 ± 0.1	0.129

Continuous data are presented as means ± standard error. **^a^** *p* < 0.05, Severe SC vs. Moderate SC; **^b^** *p* < 0.05, Severe SC vs. Mild SC; **^c^** *p* < 0.05, Moderate SC vs. Mild SC (Bonferroni’s post hoc analysis). SC = septic cardiomyopathy; aLVEF = afterload-adjusted LVEF; HR = heart rate; MAP = mean arterial pressure; SOFA = Sequential Organ Failure Assessment; cTnI = cardiac Troponin I; BNP = B-type natriuretic peptide; SVR = systemic vascular resistance; EVLW = extravascular lung water; ELWI = extravascular lung water index; ScvO_2_ = central venous oxygen saturation; CVP = central venous pressure; VTI = velocity time integral; aVTI = afterload-adjusted VTI; LVEDV = left ventricular end-diastolic volume; LVEF = left ventricular ejection fraction; E/Em = early diastolic transmitral flow velocity (E) to early diastolic mitral annular tissue velocity (Em).

## Data Availability

The datasets used and/or analyzed during the current study are available from the corresponding author on reasonable request.

## Supplementary Materials

The following supporting information can be downloaded at: https://www.mdpi.com/article/10.3390/jpm12111786/s1. Supplemental file S1. Additional details on methodology used. Supplemental Table S1. A correlation matrix illustrating correlation coefficients between echocardiographic and hemodynamic variables examined in our study.

## References

[1] Vieillard-Baron A., Millington S.J., Sanfilippo F., Chew M., Diaz-Gomez J., McLean A., Pinsky M.R., Pulido J., Mayo P., Fletcher N. (2019). A decade of progress in critical care echocardiography: A narrative review. Intensive Care Med..

[2] Noritomi D.T., Vieira M.L.C., Mohovic T., Bastos J.F., Cordioli R.L., Akamine N., Fischer C.H. (2010). Echocardiography for Hemodynamic Evaluation in the Intensive Care Unit. Shock.

[3] Oh J.K. (2005). Echocardiography as a Noninvasive Swan-Ganz Catheter. Circulation.

[4] Burgdorff A.-M., Bucher M., Schumann J. (2018). Vasoplegia in patients with sepsis and septic shock: Pathways and mechanisms. J. Int. Med. Res..

[5] Hunter J.D., Doddi M. (2010). Sepsis and the heart. Br. J. Anaesth..

[6] Vieillard-Baron A. (2011). Septic cardiomyopathy. Ann. Intensive Care.

[7] Ciampi Q., Villari B. (2007). Role of echocardiography in diagnosis and risk stratification in heart failure with left ventricular systolic dysfunction. Cardiovasc. Ultrasound.

[8] Tan C., Rubenson D., Srivastava A., Mohan R., Smith M.R., Billick K., Bardarian S., Heywood J.T. (2017). Left ventricular outflow tract velocity time integral outperforms ejection fraction and Doppler-derived cardiac output for predicting outcomes in a select advanced heart failure cohort. Cardiovasc. Ultrasound.

[9] Monnet X., Teboul J.-L. (2013). Assessment of volume responsiveness during mechanical ventilation: Recent advances. Crit. Care.

[10] Maizel J., Airapetian N., Lorne E., Tribouilloy C., Massy Z., Slama M. (2007). Diagnosis of central hypovolemia by using passive leg raising. Intensive Care Med..

[11] Lamia B., Ochagavia A., Monnet X., Chemla D., Richard C., Teboul J.-L. (2007). Echocardiographic prediction of volume responsiveness in critically ill patients with spontaneously breathing activity. Intensive Care Med..

[12] Parker M.M., Shelhamer J.H., Bacharach S.L., Green M.V., Natanson C., Frederick T.M., Damske B.A., Parrillo J.E. (1984). Profound but Reversible Myocardial Depression in Patients with Septic Shock. Ann. Intern. Med..

[13] Santos T., Schweller M., Gontijo-Coutinho C., Franci D., Nocera P., Guerra-Grangeia T., Matos-Souza J., Carvalho-Filho M. (2015). Sepsis survivors present with higher values of cardiac index and velocity time integral in the emergency department. Crit. Care.

[14] L’Heureux M., Sternberg M., Brath L., Turlington J., Kashiouris M.G. (2020). Sepsis-Induced Cardiomyopathy: A Comprehensive Review. Curr. Cardiol. Rep..

[15] Boissier F., Razazi K., Seemann A., Bedet A., Thille A.W., De Prost N., Lim P., Brun-Buisson C., Dessap A.M. (2017). Left ventricular systolic dysfunction during septic shock: The role of loading conditions. Intensive Care Med..

[16] Repessé X., Charron C., Vieillard-Baron A. (2013). Evaluation of left ventricular systolic function revisited in septic shock. Crit. Care.

[17] Bergenzaun L., Gudmundsson P., Öhlin H., Düring J., Ersson A., Ihrman L., Willenheimer R., Chew M.S. (2011). Assessing left ventricular systolic function in shock: Evaluation of echocar-diographic parameters in intensive care. Crit. Care.

[18] Dellinger R.P., Levy M.M., Rhodes A., Annane D., Gerlach H., Opal S.M., Sevransky J.E., Sprung C.L., Douglas I.S., Jaeschke R. (2013). Surviving sepsis campaign: International guidelines for management of severe sepsis and septic shock, 2012. Intensive Care Med..

[19] Moreno R., Vincent J.-L., Matos R.T., Mendonça A., Cantraine F., Thijs L., Takala J., Sprung C., Antonelli M., Bruining H. (1999). The use of maximum SOFA score to quantify organ dysfunction/failure in intensive care. Results of a prospective, multicentre study. Intensive Care Med..

[20] Horan T.C., Andrus M., Dudeck M.A. (2008). CDC/NHSN surveillance defnition of health care-associated infection and criteria for specifc types of infections in the acute care setting. Am. J. Infect. Control.

[21] Vieillard-Baron A., Caille V., Charron C., Belliard G., Page B., Jardin F. (2008). Actual incidence of global left ventricular hypokinesia in adult septic shock. Crit. Care Med..

[22] Beesley S.J., Weber G., Sarge T., Nikravan S., Grissom C.K., Lanspa M.J., Shahul S., Brown S.M. (2018). Septic Cardiomyopathy. Crit. Care Med..

[23] Baumgartner H., Hung J., Bermejo J., Chambers J.B., Evangelista A., Griffin B.P., Iung B., Otto C.M., Pellikka P.A., Quiñones M. (2009). Echocardiographic Assessment of Valve Stenosis: EAE/ASE Recommendations for Clinical Practice. J. Am. Soc. Echocardiogr..

[24] Blanco P. (2020). Rationale for using the velocity–time integral and the minute distance for assessing the stroke volume and cardiac output in point-of-care settings. Ultrasound J..

[25] Shiran A., Adawi S., Ganaeem M., Asmer E. (2008). Accuracy and reproducibility of left ventricular outflow tract diameter measurement using transthoracic when compared with transesophageal echocardiography in systole and diastole. Eur. J. Echocardiogr..

[26] Schiller N.B., Shah P.M., Crawford M., DeMaria A., Devereux R., Feigenbaum H., Gutgesell H., Reichek N., Sahn D., Schnittger I. (1989). Recommendations for quantitation of the left ventricle by two-dimensional echo-cardiography. American Society of Echocardiography Committee on Standards, Subcommittee on Quantitation of Two-Dimensional Echocardiograms. J. Am. Soc. Echocardiogr..

[27] Nagueh S.F., Smiseth O.A., Appleton C.P., Byrd B.F., Dokainish H., Edvardsen T., Flachskampf F.A., Gillebert T.C., Klein A.L., Lancellotti P. (2016). Recommendations for the evaluation of left ventricular diastolic function by echocardiography: An update from the American society of echocardiography and the European association of cardiovascular imaging. Eur. J. Echocardiogr..

[28] Pulsion Medical Systems, PiCCO2 Technical Datasheet, © PULSION EN 03/2013 March 2013 Art. No.: PC856EN_R11.

[29] Renner L.E., Morton M.J., Sakuma G.Y. (1993). Indicator amount, temperature, and intrinsic cardiac output affect thermodilution cardiac output accuracy and reproducibility. Crit. Care Med..

[30] Goldman J.H., Schiller N.B., Lim D.C., Redberg R.F., Foster E. (2001). Usefulness of stroke distance by echocardiography as a surrogate marker of cardiac output that is independent of gender and size in a normal population. Am. J. Cardiol..

[31] Bouhemad B., Nicolas-Robin A., Arbelot C., Arthaud M., Féger F., Rouby J.-J. (2009). Acute left ventricular dilatation and shock-induced myocardial dysfunction. Crit. Care Med..

[32] Huang S.J., Nalos M., McLean A.S. (2013). Is early ventricular dysfunction or dilatation associated with lower mortality rate in adult severe sepsis and septic shock? A meta-analysis. Crit. Care.

[33] Jardin F., Brun-Ney D., Auvert B., Beauchet A., Bourdarias J.P. (1990). Sepsis-related cardiogenic shock. Crit. Care Med..

[34] Ehrman R.R., Sullivan A.N., Favot M.J., Sherwin R.L., Reynolds C., Abidov A., Levy P.D. (2018). Pathophysiology, echocardiographic evaluation, biomarker findings, and prognostic implications of septic cardiomyopathy: A review of the literature. Crit. Care.

[35] Papanikolaou J., Makris D., Mpaka M., Palli E., Zygoulis P., Zakynthinos E. (2014). New insights into the mechanisms involved in B-type natriuretic peptide elevation and its prognostic value in septic patients. Crit. Care.

[36] Israel G.D. Determining Sample Size. University of Florida Cooperative Extension Service, Institute of Food and Agriculture Sciences, EDIS, Florida, FL, USA, 1992.

[37] Via G., Price S., Storti E. (2011). Echocardiography in the sepsis syndromes. Crit. Ultrasound J..

