# Supplementary material for: The Role of Left Ventricular Ejection Fraction and Left Ventricular Outflow Tract Velocity-Time Integral in Assessing Cardiovascular Impairment in Septic Shock
Source: J Pers Med. 2022 Oct 29;12(11):1786. doi: 10.3390/jpm12111786 (PMC9696803; doi:10.3390/jpm12111786)
Supplement: Supplementary file 1 [file jpm-12-01786-s001.zip › jpm-1949087-supplementary.pdf]

The role of left ventricular ejection fraction and left ventricular outflow tact velocity-time integral in assessing cardiovascular impairment in septic shock.

Konstantinos Spathoulas[1,2], Vasiliki Tsolaki[2], George E. Zakynthinos[2], Dimitrios Karelas[1], Demosthenes Makris[2], Epaminondas Zakynthinos[2], John Papanikolaou[1,2]

[1] Department of Cardiology, General Hospital of Trikala, Thessaly, Greece

[2] Department of Critical Care, School of Medicine, University of Thessaly, University Hospital of Larissa, Thessaly, Greece

Methods

Clinical assessment

Patients' clinical information at baseline included age, sex, and reason for admission. Acute Physiology and Chronic Health Evaluation II (APACHE II) score reflected the severity of disease upon admission. The Sequential Organ Failure Assessment (SOFA) score was quantified daily during the study period as a marker of the degree of organ dysfunction/failure [1]. Clinical data also included ICU infections (site and microbiology) and the noradrenaline dose at each examination.

Infections were defined according to criteria of Centers for Disease Control and prevention (Atlanta, GA, USA) [2]. Patients were routinely cultured on the onset of shock and sampling was repeated at 24-hours intervals. All patients received initially antibiotic treatment empirically until the culprit pathogen was isolated.

Patients were considered as "septic shock survivors" if they survived the episode of septic shock.

Transthoracic Echocardiography (TTE) examination and PiCCO measurements

TTE measurements

Echocardiography (Phillips iE33, Andover, MA, U.S.A.) was performed by two experienced echocardiographers (J.P., E.Z.). LVOT VTI was measured by pulsed wave Doppler transthoracic echocardiography from the apical five chamber view as recommended [3]. Efforts were made in order the beam-flow angulation at LVOT to practically approach 0° degrees [4]. During each examination, five LVOT VTI measurements were obtained at end-expiration, and the mean value was recorded. LVOT diameter ($LVOT_D$) was measured from the parasternal long axis view at mid-

systole just below the insertion of the aortic valve leaflets [5]. Calculation of SV was based on the mathematical equation: $SV = \pi*LVOT_D^2*VTI/4= 0.785*LVOT_D^2*VTI$. Echocardiographic CO was derived by the product echocardiographic SV times the heart rate (HR): CO=SV*HR. Minute distance was calculated as LVOT VTI times the HR in order to monitor CO fluctuations in the same individual without the need for serial $LVOT_D$ re-assessment [4].

LVEF as well as LV end-diastolic volume (LVEDV) values were measured by the two-dimensional Simpson's biplane method of disks, by using both apical four- and two-chamber views [6].

Mitral inflow pulsed-wave Doppler signals and tissue Doppler imaging signals at the lateral mitral annulus were also recorded. Analysis was performed for end-expiratory E/Em ratios as indices of LV filling pressures [7].

PiCCO measurements

The PiCCO system (Pulsiocath 5F, 20 cm, PV2015L20; Pulsion Medical Systems AG, Munich, Germany) was inserted by the jugular or subclavian vein and remained in place for at least three consecutive days. PiCCO provided hemodynamic data, including CO, SVR, extravascular lung water (EVLW) (along with their indexed values) and central venous pressure (CVP). The PiCCO device was carefully calibrated before starting each measurement. Patients were studied while supine, and zero pressure was measured at atmospheric pressure at the midaxillary line.

Three calculations of SV, CO and SVR through the PiCCO thermodilution method were performed by using cold saline and the mean values for each parameter were recorded. The injectate solution was 0.9% saline, as both lipid and dextrose

compounds can damage the sensor’s housing [8]. The injectate volume per injection was 20ml and the fluid temperature was below 8°C (although room temperature solutions are also acceptable) [9]. Three separate injections within a five-minute period were performed. Those thermodilution curves showing irregularities or lacking a clear early peak were considered inadequate and therefore rejected. If the difference between the lowest and highest values of the three measurements was >10%, additional cardiac output measurements were performed, and extreme values discarded. PiCCO measurements and analyses were performed by K.S., V.T., G.Z. (at least two clinicians were present at each measurement). Physicians who performed the echocardiographic examination were unaware of the results of the thermodilution study.

References

1. Moreno R, Vincent JL, Matos R, et al. The use of maximum SOFA score to quantify organ dysfunction/failure in intensive care. Results of a prospective, multicentre study. Working Group on Sepsis related Problems of the ESICM. Intensive Care Med. 1999;25(7):686-96.
2. Horan TC, Andrus M, Dudeck MA. CDC/NHSN surveillance defnition of health care-associated infection and criteria for specifc types of infections in the acute care setting. Am J Infect Control. 2008;36(5):309–32.
3. Baumgartner H, Hung J, Bermejo J, et al; American Society of Echocardiography; European Association of Echocardiography. Echocardiographic assessment of valve stenosis: EAE/ASE recommendations for clinical practice. J Am Soc Echocardiogr. 2009 Jan;22(1):1-23; quiz 101-2. doi: 10.1016/j.echo.2008.11.029. Erratum in: J Am Soc Echocardiogr. 2009 May;22(5):442.
4. Blanco, P. Rationale for using the velocity–time integral and the minute distance for assessing the stroke volume and cardiac output in point-of-care settings. Ultrasound J. 2020;12(1):21
5. Avinoam Shiran, Salim Adawi, Majdi Ganaeem, Ehab Asmer. Accuracy and reproducibility of left ventricular outflow tract diameter measurement using transthoracic when compared with transesophageal echocardiography in systole and diastole. Eur J Echocardiogr. 2009; 10(2):319-324
6. Schiller NB, Shah PM, Crawford M, et al. Recommendations for quantitation of the left ventricle by two-dimensional echocardiography. American Society of Echocardiography Committee on Standards, Subcommittee on Quantitation of Two-Dimensional Echocardiograms. J Am Soc Echocardiogr. 1989;2(5):358-367.

7. Nagueh, SF, Smiseth, OA, Appleton, CP, et al. Recommendations for the evaluation of left ventricular diastolic function by echocardiography: an update from the American society of echocardiography and the European association of cardiovascular imaging J Am Soc Echocardiogr 2016; 29: 277–314
8. Pulsion Medical Systems, PiCCO2 Technical Datasheet ref: MPI850305_R05. 2011.
9. Renner LE, Morton MJ, Sakuma GY. Indicator amount, temperature, and intrinsic cardiac output affect thermodilution cardiac output accuracy and reproducibility. Crit Care Med 1993; 21:586-597

Supplemental Table S1. A correlation matrix illustrating correlation coefficients between echocardiographic and hemodynamic examined variables in our study

	**LVEF**	**VTI**	**E/Em**	**Minute distance**	**SV (PiCCO)**	**CO (PiCCO)**	**SVR**	**EVLW**	**$ScvO_2$**	**CVP**	**aLVEF**	**aVTI**
**LVEF**												
**VTI**	0.406**											
**E/Em**	-	-0.288*										
**Minute distance**	0.605**	0.752**	-									
**SV (PiCCO)**	0.391**	0.910**	-	0.741**								
**CO (PiCCO)**	0.514**	0.743**	-	0.895**	0.892**							
**SVR**	-0.428**	-0.702**	-	-0.826**	-0.763**	0.827**						
**EVLW**	-	-	-	-	-	-	-					
**$ScvO_2$**	-	0.473**	-	0.362*	0.463**	0.391*	-0.486**	-				
**CVP**	-	-	-	-	-	-	-	-	-			
**aLVEF**	0.876**	-	-	-	-	-	-	-	-	-		
**aVTI**	-	0.691**	-0.318**	-	0.468**	-	-	-	-	0.251*	-	

Numbers express *Pearson's* correlation coefficients; ** $P<0.01$; *$0.01\leq P<0.05$

LVEF= left ventricular ejection fraction; VTI= velocity-time integral; E/Em= early diastolic transmitral flow velocity (E) to early diastolic mitral annular tissue velocity (Em); Minute distance=; SV= stroke volume; CO= cardiac output; SVR= systemic vascular resistance; EVLW= extravascular lung water; $ScvO_2$= central venous oxygen saturation; CVP= central venous pressure; aLVEF= afterload-adjusted LVEF; aVTI= afterload-adjusted VTI.
